# Vancomycin-induced gut microbiota dysbiosis aggravates allergic rhinitis in mice by altered short-chain fatty acids

**DOI:** 10.3389/fmicb.2022.1002084

**Published:** 2022-11-01

**Authors:** Zhen Chen, Qingqing Xu, Yang Liu, Yihan Wei, Shancai He, Wei Lin, Yingge Wang, Li Li, Yuanteng Xu

**Affiliations:** ^1^Department of Otorhinolaryngology Head and Neck Surgery, the First Affiliated Hospital of Fujian Medical University, Fuzhou, China; ^2^Allergy Center, the First Affiliated Hospital of Fujian Medical University, Fuzhou, China; ^3^Department of Otorhinolaryngology, Fuqing City Hospital Affiliated to Fujian Medical University, Fuzhou, China; ^4^College of Life Sciences, Fujian Normal University, Fuzhou, China

**Keywords:** allergic rhinitis, gut microbiota dysbiosis, short-chain fatty acids, butyrate, tregs

## Abstract

**Objective:**

This study aims to explore how gut microbiota dysbiosis affects allergic rhinitis (AR) and whether short-chain fatty acids (SCFAs) play a role in this process.

**Methods:**

A mouse gut microbiota dysbiosis model was established by adding vancomycin to drinking water for 2 weeks before ovalbumin (OVA) sensitization. Then an OVA-alum AR mouse model was established by intraperitoneal OVA injection followed by nasal excitation. Hematoxylin and eosin (H&E) staining was performed to observe pathological changes in nasal and colon tissues of AR mice. Serum levels of total-IgE, OVA-sIgE, IL-4, IL-5, IL-10, and TGF-β1 were measured. The composition and diversity of the mouse gut microbiota were observed by 16S rDNA sequencing. Levels of SCFAs in feces were determined using SCFA-targeted metabolomics. Sodium butyrate (NaB) was added daily to mice on a low-fiber basal diet 2 weeks before the first sensitization, until the end of the study.

**Results:**

After gut microbiota dysbiosis, serum levels of the total IgE, OVA-sIgE, IL-4, and IL-5 in AR mice were significantly increased, compared with the control group. The composition and diversity of gut microbiota were significantly altered after gut microbiota dysbiosis, with the fecal SCFAs significantly reduced as well. The reduced bacterial genera after gut microbiota dysbiosis, such as *Ruminococcus* and *Lactobacillus*, were significantly and positively correlated with SCFAs. In contrast, the increased genera in the Van group, such as *Escherichia-Shigella* and *Klebsiella,* were significantly negatively correlated with SCFAs in feces. NaB treatment significantly reduced total-IgE, OVA-sIgE, IL-4, and IL-5 levels in serum, and inflammatory infiltration of the nasal and colon mucosa. In addition, serum levels of IL-10 and TGF-β1 increased significantly after NaB treatment. Foxp3 protein in the colon was upregulated considerably after NaB intervention.

**Conclusion:**

Vancomycin-induced gut microbiota dysbiosis increased susceptibility and severity of AR, which is significantly related to reduced SCFA-producing bacteria, fecal SCFAs, and specific bacterial taxa. In addition, it was found that NaB alleviated low dietary fiber base-fed symptoms and immune status in AR mice.

## Introduction

Allergic rhinitis (AR) is nasal inflammation caused by an excessive immune response to allergens (Greiner et al., 2011). The incidence rate of AR worldwide is estimated between 10% and 30% and is increasing (Wheatley and Togias, 2015). AR is caused by an interaction of genetic and environmental factors. According to the hygiene hypothesis, an increase in the incidence of AR is associated with changes in environmental factors such as reduced colonization of gut microbiota during early life, early-life antimicrobial exposure (Johnson et al., 2005), cesarean birth (Renz-Polster et al., 2005), formula feeding (Friedman and Zeiger, 2005), and lack of maternal exposure to pets or livestock during pregnancy (Ownby et al., 2002). In general, low richness and diversity of the gut microbiota have been linked to the development of allergic diseases. Recently, epidemiological studies have provided evidence for a possible relationship between gut microbiota dysbiosis and the risk of AR (Chua et al., 2018; Su et al., 2021; Zhou et al., 2021). In addition, numerous studies have reported perturbations in the balance of gut microbiota are correlated with the development and progression of allergic and airway hyperresponsiveness diseases (Cait et al., 2018; Langan et al., 2020; Zhang et al., 2021; Huang et al., 2022).

Previous research has found vancomycin dramatically alters the mouse gut microbiome, leading to disrupted immune homeostasis and increased susceptibility to allergic asthma (Russell et al., 2012). Disruption of the microbiome after antibiotic treatment results in an altered metabolome with attenuated production of short-chain fatty acids (SCFAs; Kim et al., 2020). Mouse early-life disruption of the microbiome *via* antibiotic treatment results in a modified metabolome with attenuated production of SCFAs, which may play a crucial role in atopic dermatitis (AD; Pascal et al., 2018). SCFAs are a product of the fermentation of dietary fiber by specific microbes and have beneficial effects on both intestinal epithelial and immune cells (Li et al., 2022), which alleviate allergic disease (Corrêa-Oliveira et al., 2016). For example, SCFAs were previously shown to play a role in modulating mouse immune response in allergic asthma (Maslowski et al., 2009; Trompette et al., 2014). In addition, Roduit et al. (2019) showed that children with the highest butyrate levels were less likely to be diagnosed with AR. Recent studies have shown that oral administration of SCFAs (Roduit et al., 2019) or butyrate (Wang et al., 2016, 2020) to mice significantly reduced the severity of allergic airway inflammation, including AR.

Previous studies have involved the correlation between intestinal microbial diversity/fecal SCFAs changes and AR, but they are not thorough enough. A recent study (Zhou et al., 2021) comprehensively used 16S rDNA sequencing and SCFA-targeted metabonomics technology to show that concentrations of fecal SCFAs were significantly lower in the AR group than in the healthy control group, and *Eubacterium-hallii* and *Blautia* were positively correlated with SCFAs, which indicates that changes in composition and function of gut microbiome in AR. SCFAs, especially butyrate, are considered a potential anti-allergenic substance, which may be one of the critical substances of gut microbiota affecting AR. However, how gut microbiota dysbiosis affects AR and whether SCFAs play a role in this process are still unclear. The combination of microbiome and metabolome will show us more relevant information, which is a reliable method to study gut microbiota and its function. However, population studies of metabolomics and 16S rDNA sequencing are significantly influenced by age, sex, and diet. In addition, it is difficult to conduct more in-depth mechanistic studies, such as vancomycin intervention, butyrate feeding, and intestinal mucosa study. Therefore, a vancomycin-induced gut microbiota dysbiosis AR mouse model was established, which overcame the above limitations.

In the present study, by establishing a gut microbiota dysbiosis AR model and using multi-omics strategies, we reported that vancomycin-induced gut microbiota dysbiosis increased susceptibility and severity of AR, which may be related to reduced SCFA-producing bacteria, fecal SCFAs, and specific bacterial taxa. In addition, by establishing an AR mice model fed a low dietary fiber basal diet or NaB, we found that NaB alleviated low dietary fiber base-fed symptoms and immune status in AR mice. This work may illuminate strategies for preventive measures and/or treatments of AR in the future.

## Materials and methods

### Animals

Fifty-two specific pathogen-free (SPF) female BALB/c mice (4 weeks old) were purchased from Shanghai Shrek experimental animals Co., Ltd. and were raised under specific pathogen-free conditions in the experimental animal center of Fujian Medical University and were maintained on a 12-h daylight cycle with access to commercial pelleted food and water *ad libitum*. All animal experiments were approved by the Institutional Animal Care and Use Committee (IACUC) of Fujian Medical University (IACUC number: FJMUIACUC2020-0106).

### Establishment of vancomycin-induced gut microbiota dysbiosis AR model

**Figure 1:**
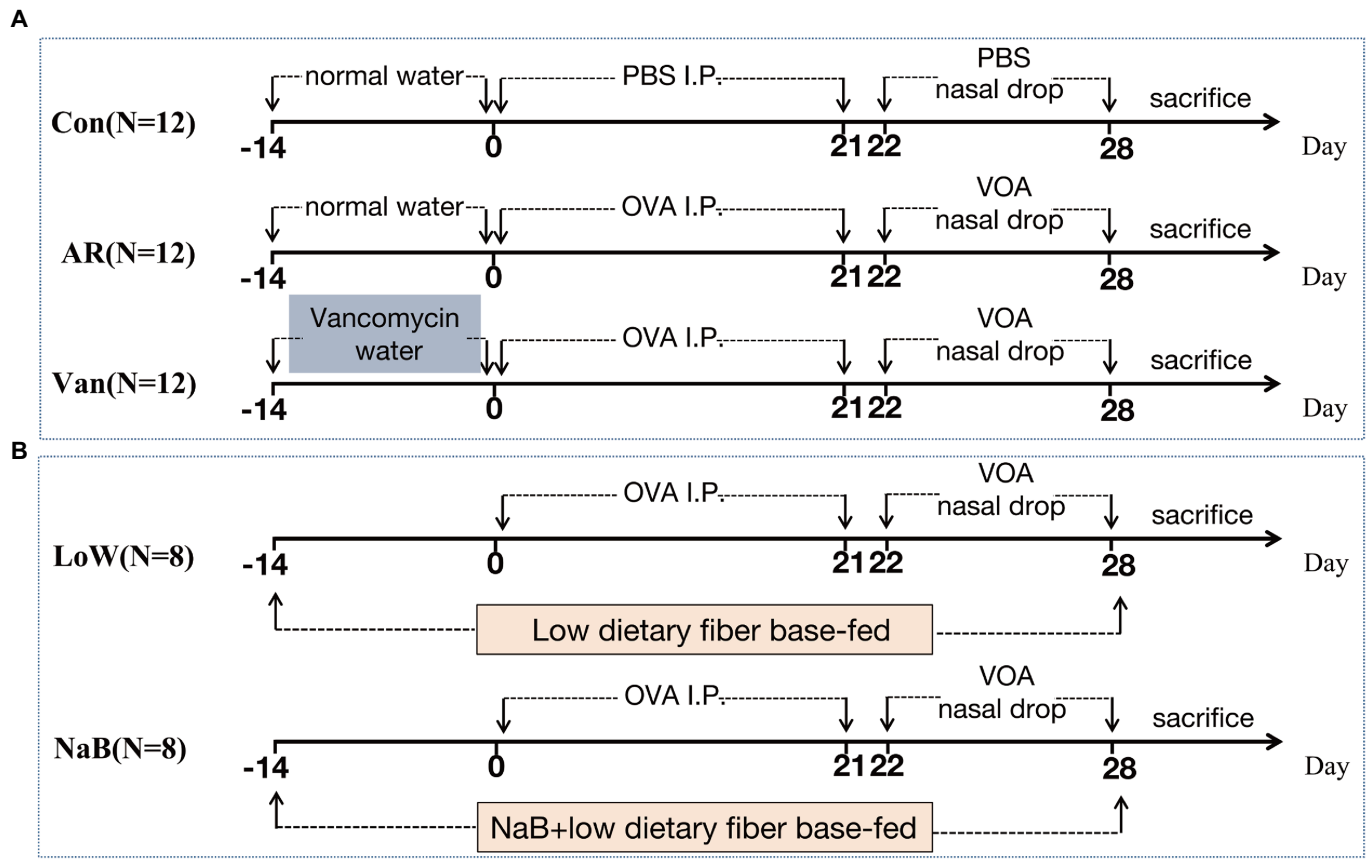
The protocol of treatment of mice in each group. **(A)** Establishment of Vancomycin-induced gut microbiota dysbiosis AR model. **(B)** Establishment of AR mice model fed a low dietary fiber basal diet or Sodium Butyrate (NaB).

Thirty-six SPF female BALB/c mice (4 weeks old) were randomly divided into three groups (*N* = 12): the control group (Control), the ovalbumin (OVA)-induced AR model group (AR), and the OVA-induced AR model plus Vancomycin (Van). For inducing gut dysbiosis, four-week-old mice of the Van group were exposed to vancomycin in drinking water (200 mg/l vancomycin hydrochloride MCE, HY-17362) for 2 weeks before primary abdominal OVA sensitization, and then water was replaced with normal water. The OVA-induced AR model was established according to previous literature with a few modifications (Cho et al., 2019). Briefly, 6-week-old mice were intraperitoneally injected with 0.2 ml of sensitization solution (40 μg OVA and 4 μg Imject™ Alum Adjuvant in 100 μl of PBS) on days 0, 7, 14, and 21. Then 10 μl of stimulation solution (100 μg OVA in 10 μl of PBS) was administered bilaterally *via* pipette into nasal cavities every day, on days 22–28. A flow chart with time coordinate and treatment of this study design is shown in Figure 1A.

### Establishment of AR mice model fed a low dietary fiber basal diet or sodium butyrate

Sixteen SPF female BALB/c mice (4 weeks old) were randomly divided into two groups (*N* = 8): the OVA-induced AR model plus low dietary fiber (Low) and the OVA-inducted AR model plus sodium butyrate (NaB). The premixed mouse feed, 30 g/kg NaB (Solarbio, S9491), was added to the low dietary fiber feed (the content of dietary fiber is about 4%) and granules were produced. Low dietary fiber feed and low dietary fiber feed plus NaB were provided by the Xiao Shu You Tai(Beijing)Biotechnology Co., Ltd. (Supplementary Table 1). Each mouse in the NaB group consumed an average of 5 g low dietary fiber feed plus NaB feed/day, which contained 0.15 g NaB, for 2 weeks before primary sensitization until the study ended. The mice of the Low group were fed a low dietary fiber diet at the same time point and amount. The establishment method of the AR mouse model is the same as above. A flow chart with time coordinate and treatment of this study design is shown in Figure 1B.

### Symptom score

After the last intranasal allergen challenge on day 28, the quantity of sneezing and nose rubbing bouts were counted for 30 min to evaluate allergic responses. Symptom scores were evaluated by a symptom scale by observers, in addition, studies were blinded to the observers (Supplementary Table 2). A total score great than five points indicated that the AR model was successfully established (Wang et al., 2011, 2020).

### Sample collection

After the last intranasal allergen challenge on day 28, blood was drawn from the ophthalmic artery, serum was extracted by centrifugation at 4,000 rpm (4°C for 10 min) and stored at −80°C. At the first time after mouse sacrifice, we cut off the colon specimen in the sterile operation and collected the feces on it in the sterile tube. And then, we repeatedly wash colon specimens with sterilized water until there is no excess material left. We divided the colon specimen into two parts on average, one part was placed in 4% formaldehyde solution for H&E staining, and the other part was placed in a refrigerator at −80° C for Western blot. The muscle tissue of the nose was removed, and the nasal cavity was fixed with 4% formaldehyde solution for 24 h at 37°C.

### H&E staining of nasal and colon tissue

The nasal and colon tissue was decalcified with a JYBL-I decalcification solution and were embedded in paraffin, and 4-μm sections were cut. The sections (4-μm) were dewaxed, stained with hematoxylin (cat. no. 245880; Abcam) for 10 min, differentiated with 1% hydrochloric acid ethanol for 1 min, stained with eosin for 1 min, dehydrated with a series of ethanol concentrations (70%, 80%, 90%, and 100%) ethanol for 10 s, incubated with xylene for 1 min and sealed. In addition, other sections were dewaxed, soaked with 3% acetic acid for 3 min, stained with 1% Alcian blue (cat. no. 150680; Abcam) for 30 min, soaked with 3% acetic acid for 3 min, washed with water, oxidized with 0.5% periodate for 10 min, soaked in Schiff’s solution for 20 min and sealed.

### Determination of serum cytokine

Serum levels of total IgE, OVA-sIgE, IL-4, IL-5, IL-10, and TGF-β1 were measured by enzyme-linked immunosorbent assay (ELISA) kits (Mskbio), according to the manufacturer’s instructions. Triple pore detection was used to calculate mean values.

### Western blot of colon tissue

Tissue lysates were made in a radioimmunoprecipitation assay (RIPA) buffer containing 25 mM Tris HCl (pH 7.2), 0.15 M NaCl, 0.1% SDS, 1% Triton X-100, 1% sodium deoxycholate, and 1 mM EDTA. Determination of protein concentration was carried out by a bicinchoninic acid protein assay kit (Pierce). After being subjected to sodium dodecyl sulfate-polyacrylamide gel electrophoresis, proteins were transferred to a nitrocellulose membrane. After blocking, total protein or phosphorylation was detected using a goat polyclonal antibody against a rabbit or mouse. Protein bands were quantified using a digital imaging system (UVtec).

### Gas chromatography–mass spectrometer analysis

20 mg of fecal samples were accurately weighed and placed in a 2 ml EP tube. 1 ml of phosphoric acid (0.5% v/v) solution and a small steel ball were added to the EP tube. The mixture was shaken vigorously for 10 s, three times, vortexed for 10 min, and ultrasonicated for 5 min. 0.1 ml of supernatant was added to 1.5 ml tubes after the mixture was centrifuged at 12,000 r/min for 10 min at 4°C. 0.5 ml MTBE (containing internal standard) solution was added to the tube. The mixture was vortexed for 3 min and ultrasonicated for 5 min. After that, the mixture was centrifuged at 12,000 r/min for 10 min at 4°C. The supernatant was collected and used for Gas chromatography–mass spectrometer (GC–MS) analysis (Bianchi et al., 2011).

An Agilent 7890B gas chromatograph coupled to a 7000D mass spectrometer with a DB-FFAP column (30 m length × 0.25 mm i.d. × 0.25 μm film thickness, J&W Scientific, United States) was employed for GC–MS/MS analysis of SCFAs. Helium was used as carrier gas at a flow rate of 1.2 ml/min. The injection was made in the split mode and the injection volume was 2 μl. The oven temperature was held at 90°C for 1 min, raised to 100°C at a rate of 25°C/min, then raised to 150°C at a rate of 20°C/min, held for 0.6 min, raised to 200°C at a rate of 25°C/min, and held for 0.5 min, after running for 3 min. All samples were analyzed in multiple reaction monitoring modes. The injector inlet and transfer line temperatures were 200°C and 230°C, respectively.

### 16S rDNA high-throughput sequencing

16S rDNA high-throughput sequencing is an important method to study the composition and structure of the microbial community in the intestine. Universal primers for PCR amplification using conserved sequences were used, and then sequencing analysis and strain identification of V3–V4 hypervariable regions was performed. Total genomic DNA was extracted from fecal samples using the CTAB/SDS method. 16S rDNA was amplified using a library of barcode-linked primers. After PCR amplification, the mixture of PCR products was purified with an AxyPrepDNA Gel Extraction Kit (AXYGEN). Sequencing libraries were generated using NEB Next®Ultra™DNA Library Prep Kit for Illumina (NEB, United States), following the manufacturer’s recommendations. Index codes were added, and the library was sequenced on an Illumina Miseq/HiSeq2500 platform, generating 250/300 bp paired-end reads. The analysis and statistics of the metagenomic data are described in supporting information.

### Statistical analysis

The data from the experiments were expressed as mean values ± standard deviation. An unpaired Student’s *t*-test was used to identify differences between two groups. Statistical significance from different groups of mice was calculated by one-way ANOVA (>2 groups). Correlation analysis of gut microbiota and fecal metabolites was evaluated using a Spearman correlation analysis. A *p*-value < 0.05 was considered to be statistically significant. Analyses were performed using SPSS version 25.0 (SPSS, Inc.) and R.

## Results

### Vancomycin-induced gut microbiota dysbiosis aggravates the severity of AR

**Figure 2:**
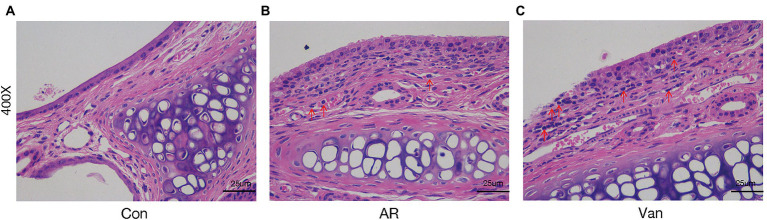
Vancomycin aggravates inflammatory cell infiltration and mucus secretion of nasal mucosa in the OVA-induced AR model. H&E staining of the nasal mucosa (400×). **(A)** the Control group; **(B)** the AR group; **(C)** the Van group.

**Figure 3:**
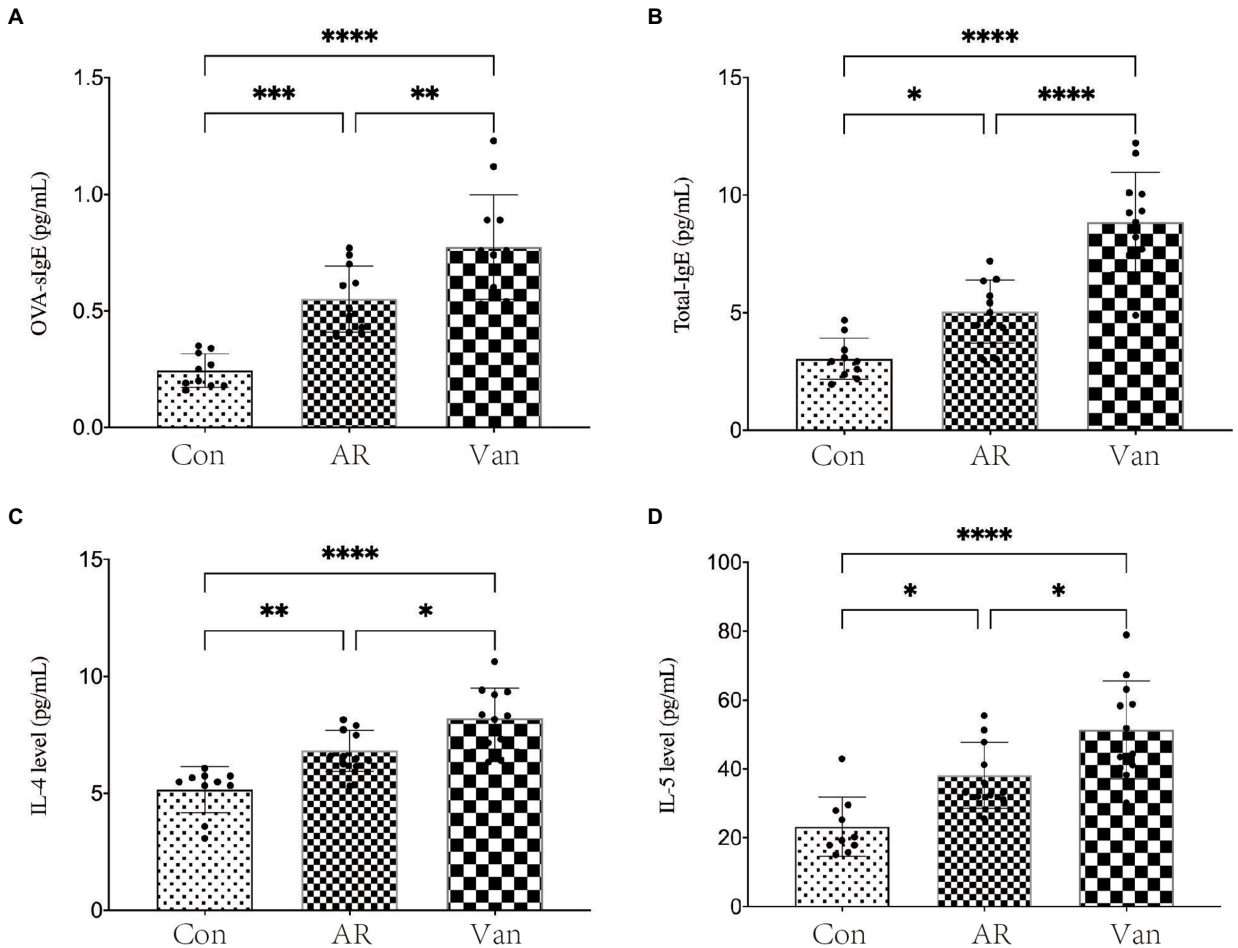
Vancomycin elevated serum cytokine and IgE levels in the OVA-induced AR model. **(A)** ELISA detection of OVA-sIgE in serum. **(B)** ELISA detection of total IgE in serum. **(C)** ELISA detection of IL-4 in serum. **(D)** ELISA detection of IL-5 in serum. ^∗^*p* < 0.05, ^∗∗^*p* < 0.01, ^∗∗∗^*p* < 0.001, ^∗∗∗∗^*p* < 0.0001.

Before sensitization, mice were given an antibiotic treatment to induce gut microbiota dysbiosis. Compared with the AR group, the mice of the Van group had aggravated nasal scratching symptoms, sparse hair around the nose, redder skin around the nose, and significantly increased nasal symptom scores (Supplementary Figure 1). Also, H&E staining further showed the nasal mucosa of the Van group mice underwent characteristic changes such as disordered arrangement of epithelial cells, partial abscission, thickening of the basement membrane, and infiltration of submucosal eosinophils (Figure 2). The above phenomena show that gut microbiota dysbiosis aggravates the symptoms of AR. IL-4 plays an essential role in the early stage of Th2 cell differentiation and is the key to triggering IgE class switching. IL-5 promotes the accumulation of eosinophils in the gut in food allergies and exacerbates eosinophil-mediated inflammatory responses. The OVA-sIgE (Figure 3A) and total IgE (Figure 3B) in the Van group were significantly increased as compared to the AR group. Compared with the AR group, IL-4 (Figure 3C) and IL-5 (Figure 3D) were significantly increased in the Van group. In summary, based on an evaluation of the nasal symptom scores, levels of serum IL-4, IL-5, total IgE, OVA-sIgE, and H&E staining of the nasal mucosa, this study shows that vancomycin treatment enhances severity of the experimental AR mouse model.

### Vancomycin treatment profoundly alters gut microbiota

**Figure 4:**
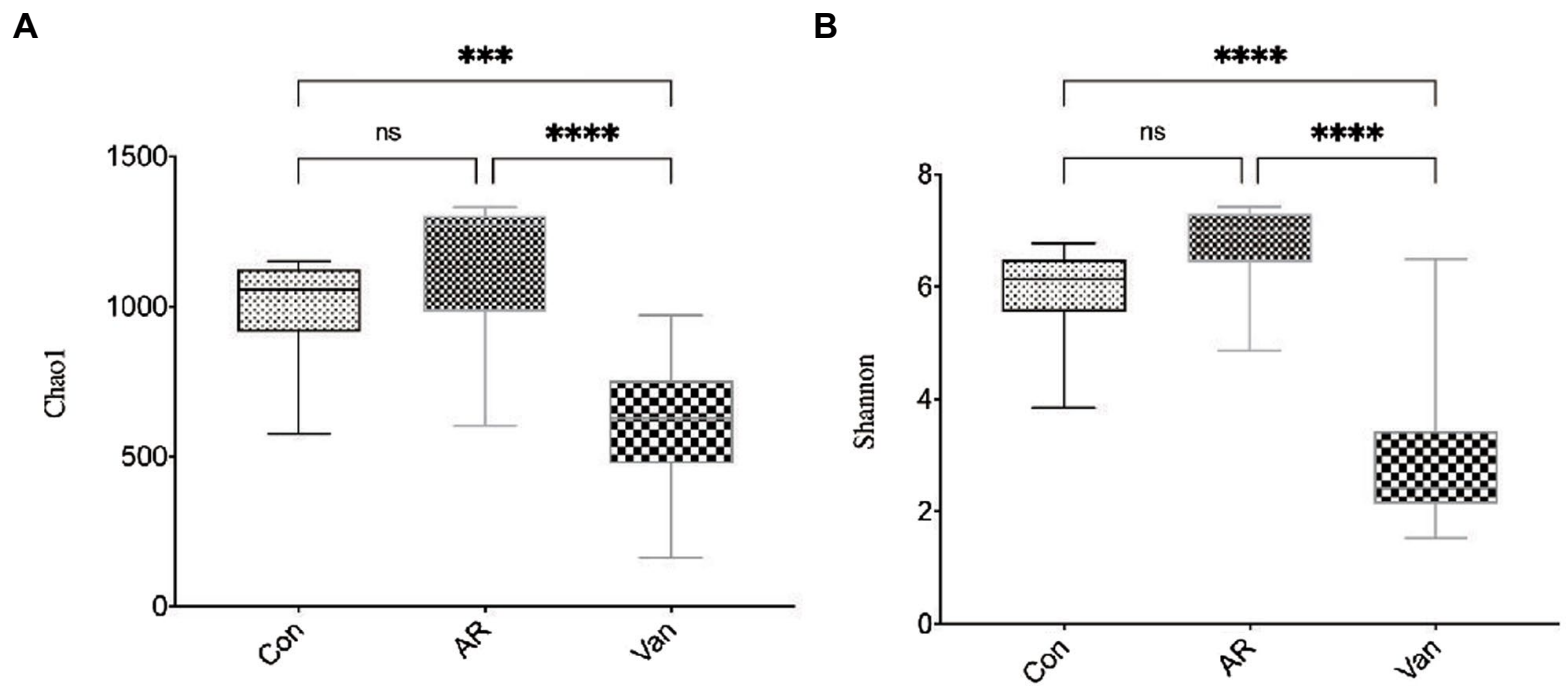
Vancomycin reduced the richness and diversity of gut microbiota in AR mice. Chao1 index **(A)** and Shannon index **(B)** at the overall level of OUT; ^∗∗∗^*p* < 0.001, ^∗∗∗∗^*p* < 0.0001.

**Figure 5:**
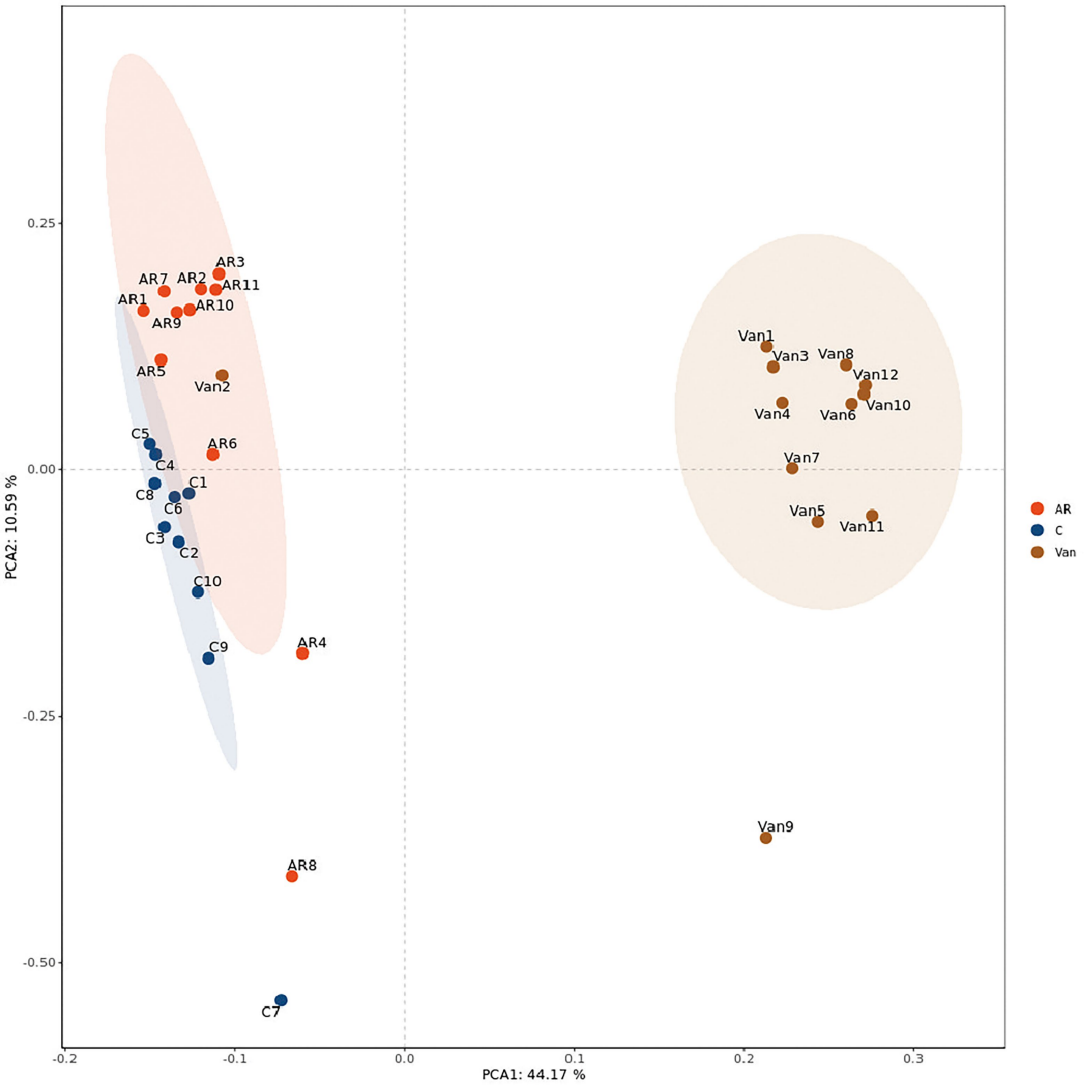
PCA analysis plot. The dots with different colors represent different sample grouping. The abscissa represents the first principal component, the ordinate represents the second principal component, and the percentage represents the contribution value to the samples difference. The more similar the community composition of the sample is, the closer their distances in the PCA map are. C, the control group; AR, the AR group; Van, the Van group.

Whether a correlation existed between increased sensitivity to AR and alterations in bacterial communities in the gut was sought after next. Total bacteria in stool pellets from antibiotic-treated mice were moderately reduced compared with the controls. The Chao1 index reflects the microbial community richness in this experiment, while the Shannon index reflects the species diversity. It was found that vancomycin intervention significantly reduced the Chao1 (Figure 4A) and Shannon (Figure 4B) index of gut microbiota in the Van group compared to the AR group. Principal coordinate analysis (PCA) was used to assess differences between the groups. The PCA plot shows that gut microbiota among the three groups is distinguished significantly (Figure 5).

**Figure 6:**
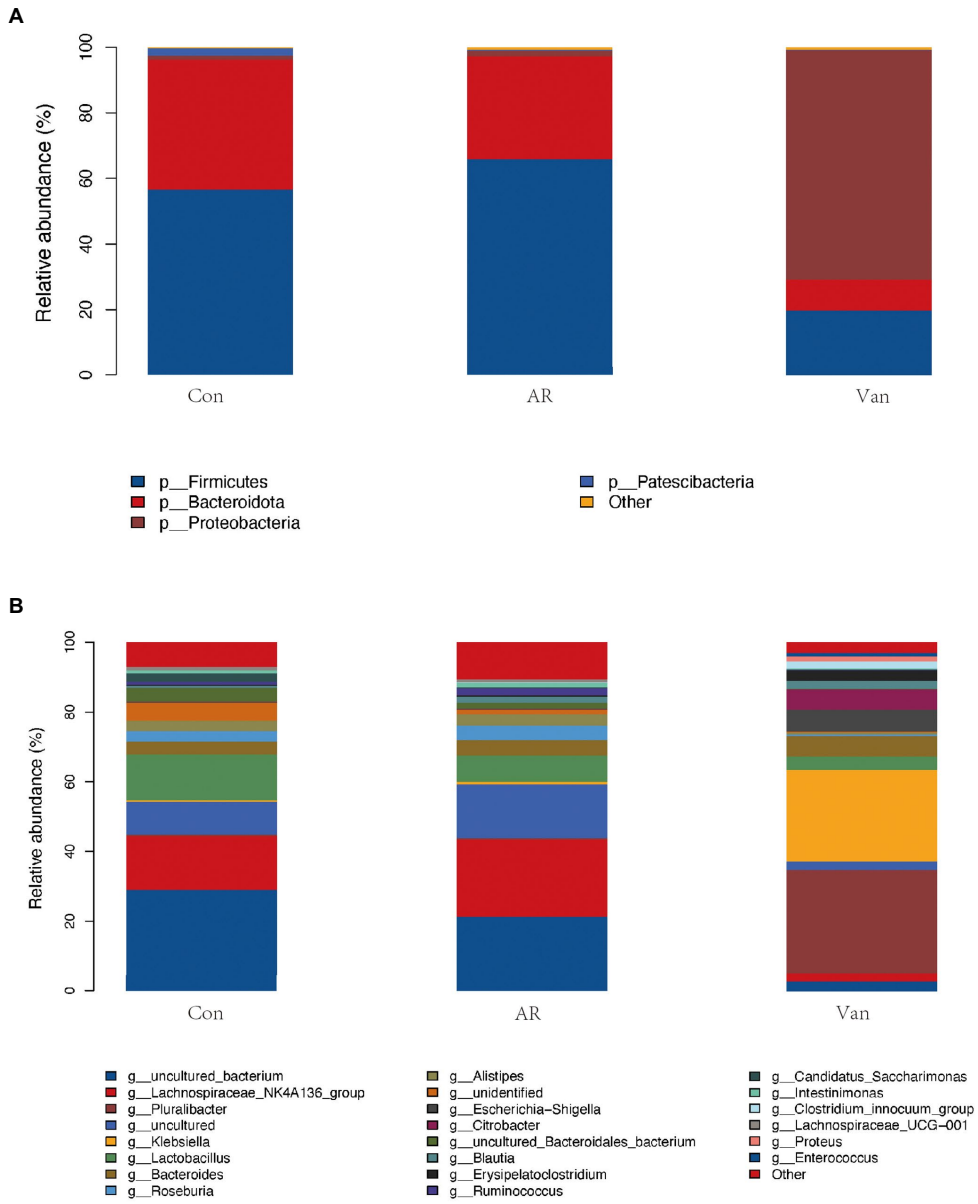
Vancomycin-induced gut microbiota dysbiosis altered the proportion of gut microbiota in the OVA-induced AR model. **(A)** Histogram of species composition analysis at the phylum level. **(B)** Histogram of species composition analysis at the genus level.

The phylum level of the gut microbiota is shown in Figure 6A. Firmicutes and Bacteroidetes accounted for more than 90% and were the dominant component in the control and AR group, followed by Proteobacteria. Vancomycin intervention significantly reduced the abundance of Firmicutes and Bacteroidetes, and the dominant bacteria group was Proteobacteria in the Van group. The proportion of Firmicutes/Bacteroidetes in the gut microbiota of the AR group was higher than that of the control group, and the proportion of Firmicutes/Bacteroidetes in the Van group was higher than that of the AR group.

The genus level of the gut microbiota is shown in Figure 6B. The main dominant bacterial genera in the Control and AR group were *g-Lachnospiraceae*_NK4A136, *g-Lactobacillus*, *g-Bacteroides*, *g-Roseburia*, and *g-Ruminococcus*, etc. (the contents of these bacteria were different between the two groups). These genera were significantly reduced in the Van group (*p* < 0.05). The dominant bacteria in the gut microbiota dysbiosis group were *g-Pluralibacter*, *g-Klebsiella*, *g-Escherichia-Shigella*, *g-Citrobacter*, etc.

**Figure 7:**
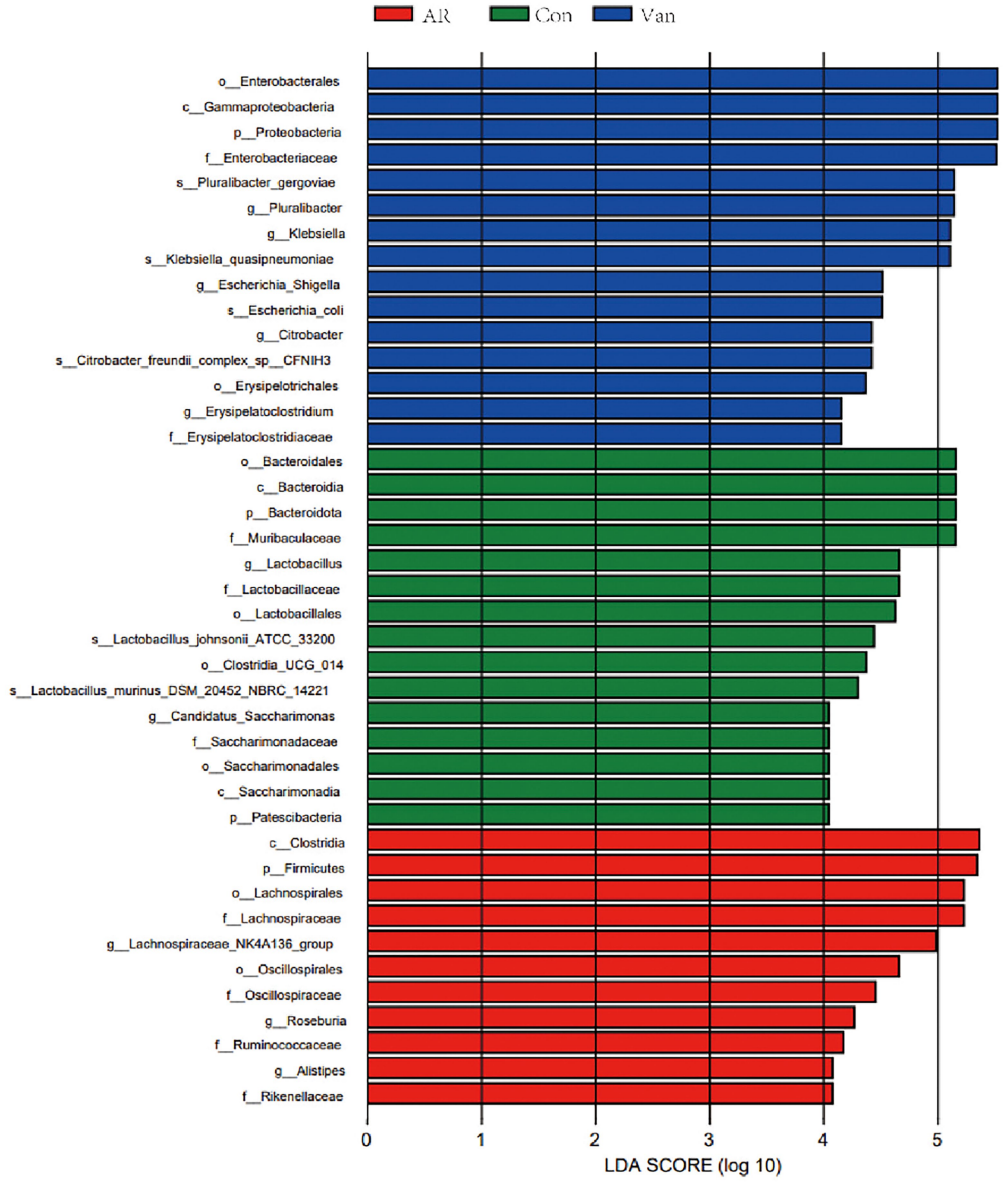
Discriminative taxa among the three groups. LDA (Linear discriminant analysis) image shows the differential bacterial genus in each group. The length of the column represents the effect of differential species on this sample (an LDA effect value of more than 4).

In this experiment, Lefse analysis was used to distinguish the Control, AR, and Van groups by identifying gut microbes at different taxonomic levels and estimating the effect size of each differentially rich microbiota. In this study, the linear discriminant analysis (LDA) was set to 4.0, and the species with LDA > 4.0 represent the key species. As shown in Figure 7, a substantial difference in the relative abundance of gut microbiota among the three groups from the phylum to the genus level is shown. The relative abundance of p-Bacteroidota, p-Patescibacteria, c-Bacteroidia, c-Saccharimonadia, o-Bacteroidales, and o-Lactobacillales in the control group increased significantly. The relative abundance of p-Firmicutes, c-Clostridia, o-Oscillospirales, o-Lachnospirales, and *f-Lachnospiraceae* in the AR group increased significantly as compared to control. The relative abundance of p-Proteobacteria, c-Gammaproteobacteria, o-Enterobacterales, and o-Erysipelotrichales rose significantly in the Van group as compared to the AR group. The greater the LDA score, the more significant the impact of the relative abundance of gut microbiota on the different effects among the three groups. The above results indicate that the vancomycin-induced gut microbiota dysbiosis model was successfully established and revealed antibiotic-specific microbial indicators of AR.

### SCFA-targeted metabolomics reveals reduced fecal SCFAs in the presence of dysbiosis of the gut microbiota

**Figure 8:**
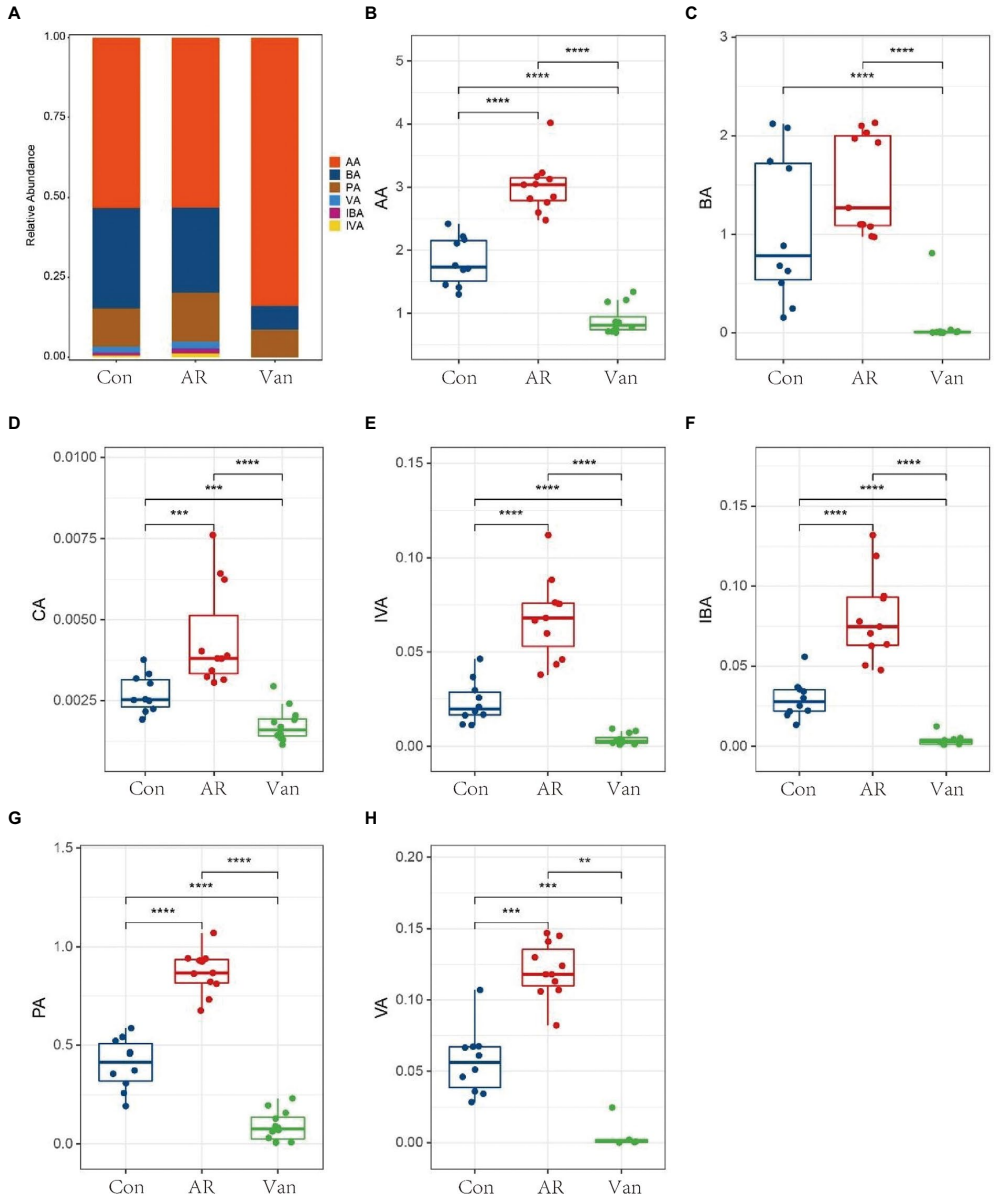
Determination of short-chain fatty acids in mouse feces based on metabolomics. **(A)** Histogram shows the average proportion value of short-chain fatty acids, **(B–H)** changes in short-chain fatty acids in feces of mice in the control group (Con), AR model group (AR), and vancomycin-induced gut microbiota dysbiosis AR model group (Van). ^**^p < 0.01, ^***^p < 0.001, ^****^p < 0.0001.

To investigate whether vancomycin-induced gut microbiota dysbiosis affects fecal SCFA levels in the gut, they were measured by GC–MS. SCFAs in the feces of mice were mainly composed of acetic acid (AA), butyrate (BA), and propionic acid (PA). Isobutyric acid (IBA), isovaleric acid (IVA), valeric acid (VA), and caproic acid (CA) accounted for only a small fraction. Vancomycin-induced gut microbiota dysbiosis AR mice exhibited significant suppression of all SCFA levels, including butyrate (Figure 8). Interestingly, compared with the control group, the amount of acetic acid, propionic acid, isobutyric acid, isovaleric acid, valeric acid, and caproic acid in the feces of the AR group were significantly increased. These data demonstrate that vancomycin-induced gut microbiota dysbiosis causes decreases in fecal SCFAs.

### Correlation analysis between fecal SCFAs and gut microbiota

**Figure 9:**
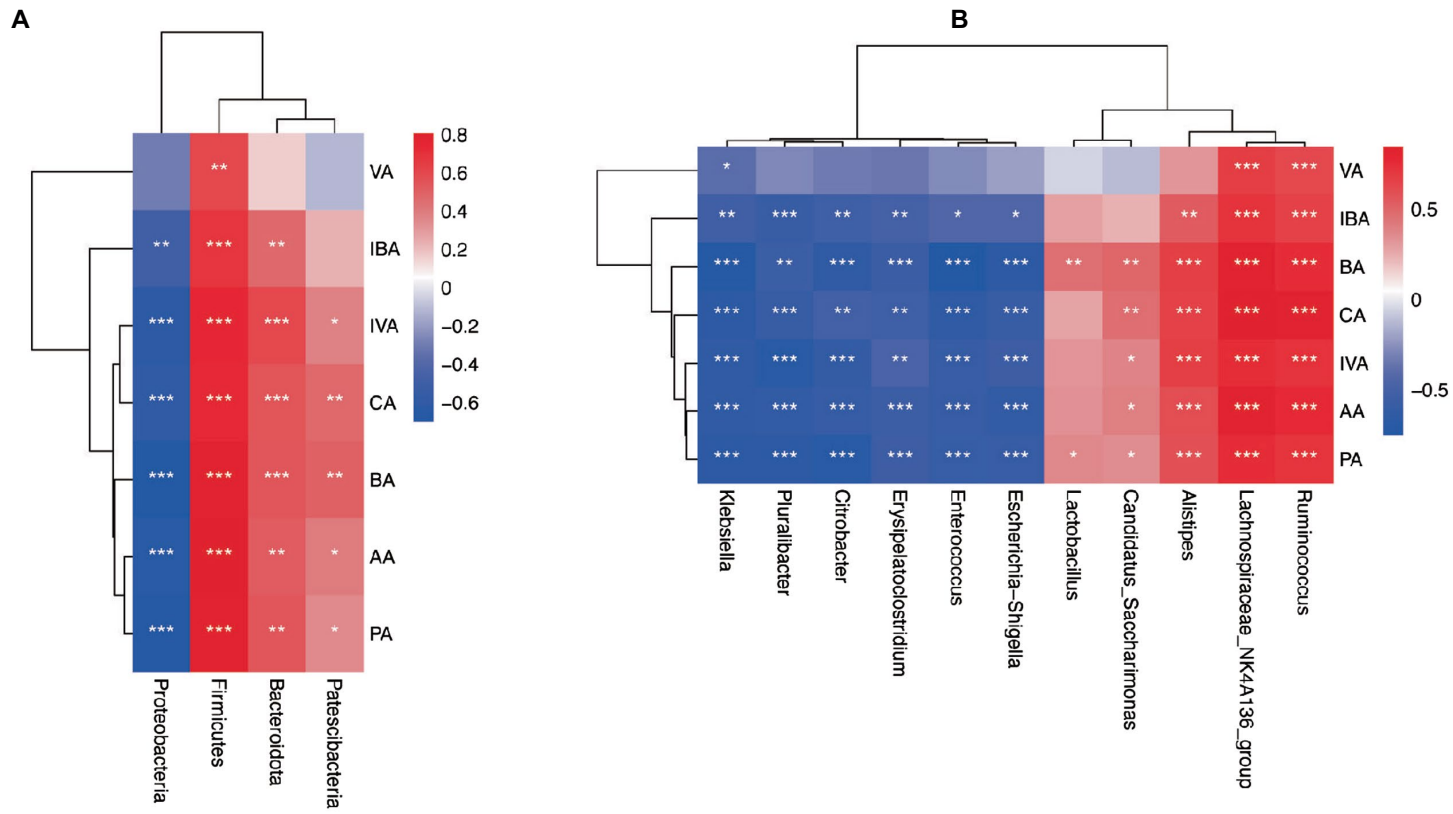
Correlation analysis heatmap. **(A)** Heat map of association between differential phyla and SCFAs. **(B)** Heat map of association between differential genera and SCFAs. acetic acid (AA), butyrate (BA), and propionic acid (PA), Isobutyric acid (IBA), isovaleric acid (IVA), valeric acid (VA), and caproic acid (CA). ^*^p < 0.05, ^**^p < 0.01, ^***^p < 0.001.

The correlation between fecal SCFAs and different gut microbiota at the phylum and genus levels was tested. Firmicutes had a significant positive correlation with acetic acid, butyrate, isobutyric acid, propionic acid, isovaleric acid, valeric acid, and caproic acid at the phylum level. Bacteroides was positively correlated with acetic acid, butyrate, isobutyric acid, propionic acid, isovaleric acid, and caproic acid. The relative abundances of Proteobacteria were significantly negatively correlated with acetic acid, butyrate, isobutyric acid, propionic acid, isovaleric acid, and caproic acid (Figure 9A).

At the genus level, the relative abundance of *Lachnospiraceae*_ NK4A136, *Ruminococcus*, and *Roseburia* (belonging to Firmicutes) were significantly positively correlated with a variety of SCFAs such as acetic acid, propionic acid, and butyrate. However, the relative abundance of *Enterococcus* and *Erysipelatoclostridium* (subordinate to Firmicutes) were both significantly negatively correlated with acetic acid, propionic acid, and butyrate. The relative abundance of *Alistipes* (subordinate to Bacteroidetes) flora was significantly positively correlated with SCFAs such as acetic acid, propionic acid, and butyrate in feces. In addition, the relative abundance of *Klebsiella*, *Pluralibacter*, *Citrobacter*, and *Escherichia-Shigella* (belonging to Proteobacteria) were all significantly negatively correlated with various SCFAs such as acetic acid, propionic acid, and butyrate (Figure 9B).

### Vancomycin-induced gut microbiota dysbiosis aggravates intestinal barrier inflammation and injury

**Figure 10:**
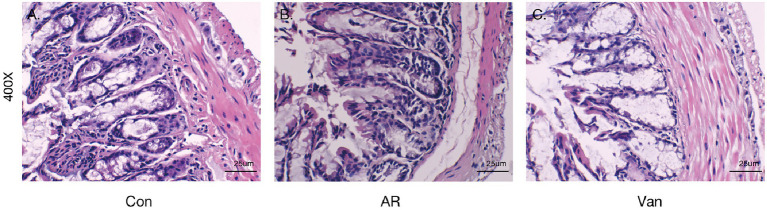
Vancomycin-induced gut microbiota dysbiosis aggravates intestinal barrier inflammation and injury. H&E staining of colonic mucosa (400×). **(A)** the Control group; **(B)** the AR group; **(C)** the Van group.

Intestinal barrier changes *via* H&E staining in the vancomycin-induced dysbiosis of intestinal microbiota in the AR model were observed (Figure 10). The colon tissue of mice in the control group exhibited normal histological features and displayed a complete villous structure. However, the intestinal tissues of mice in the other two groups were damaged. The colonic mucosa of AR mice was injured obviously accompanied by mucosal thinning. Mice in the Van group showed more severe colonic mucosa injury and inflammatory cell infiltration, with the intestinal mucosal epithelial cells atrophied and desquamated.

### Butyrate may alleviate symptoms in AR mice fed a low dietary fiber basal diet

**Figure 11:**
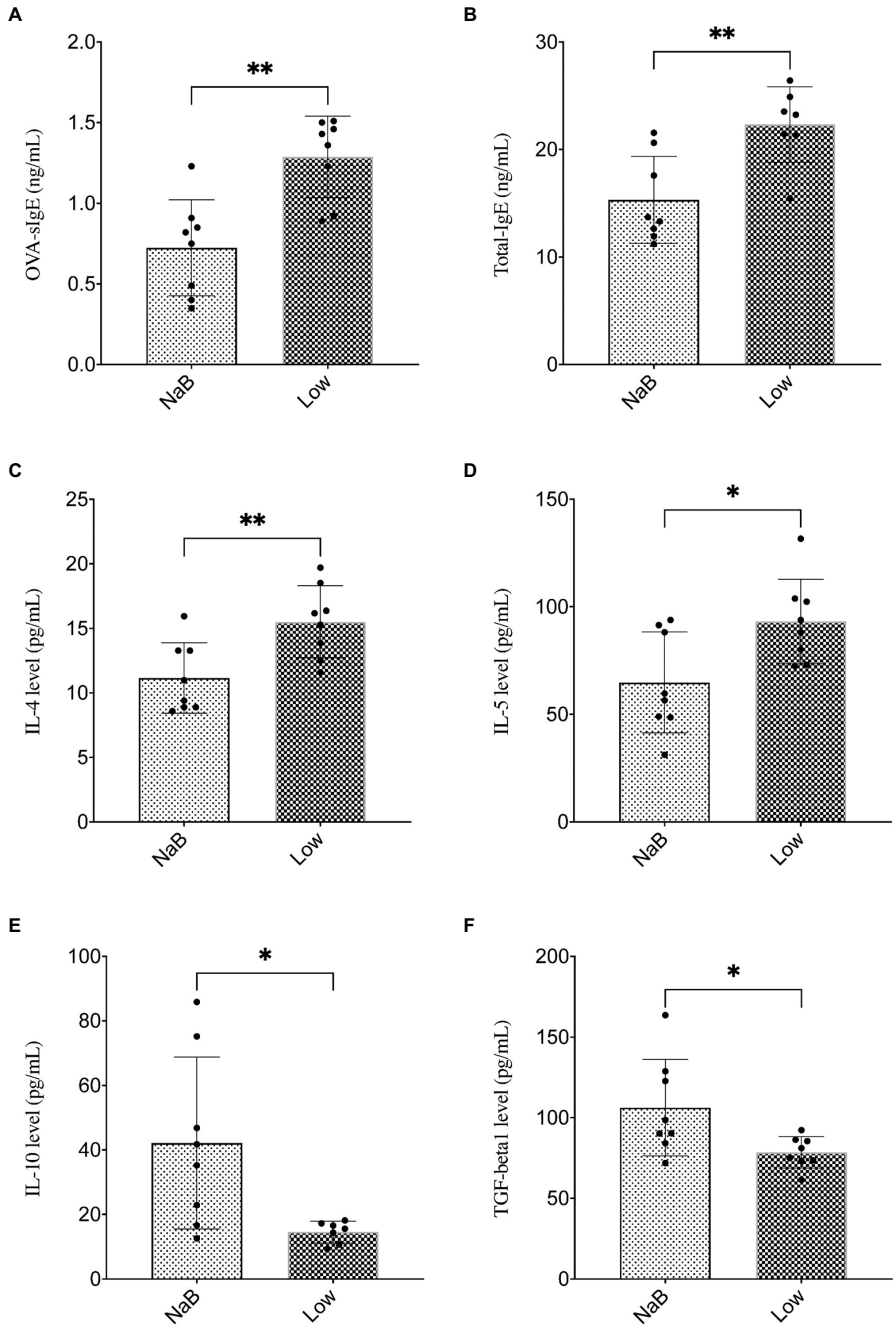
Butyrate alleviated symptoms in AR mice fed a low dietary fiber basal diet. **(A,B)** ELISA detection of OVA-sIgE and total IgE in serum. **(C–F)** ELISA detection of serum cytokines (IL-4, IL-5, IL-10, and TGF-β1). ^∗^*p* < 0.05, ^∗∗^*p* < 0.01.

**Figure 12:**
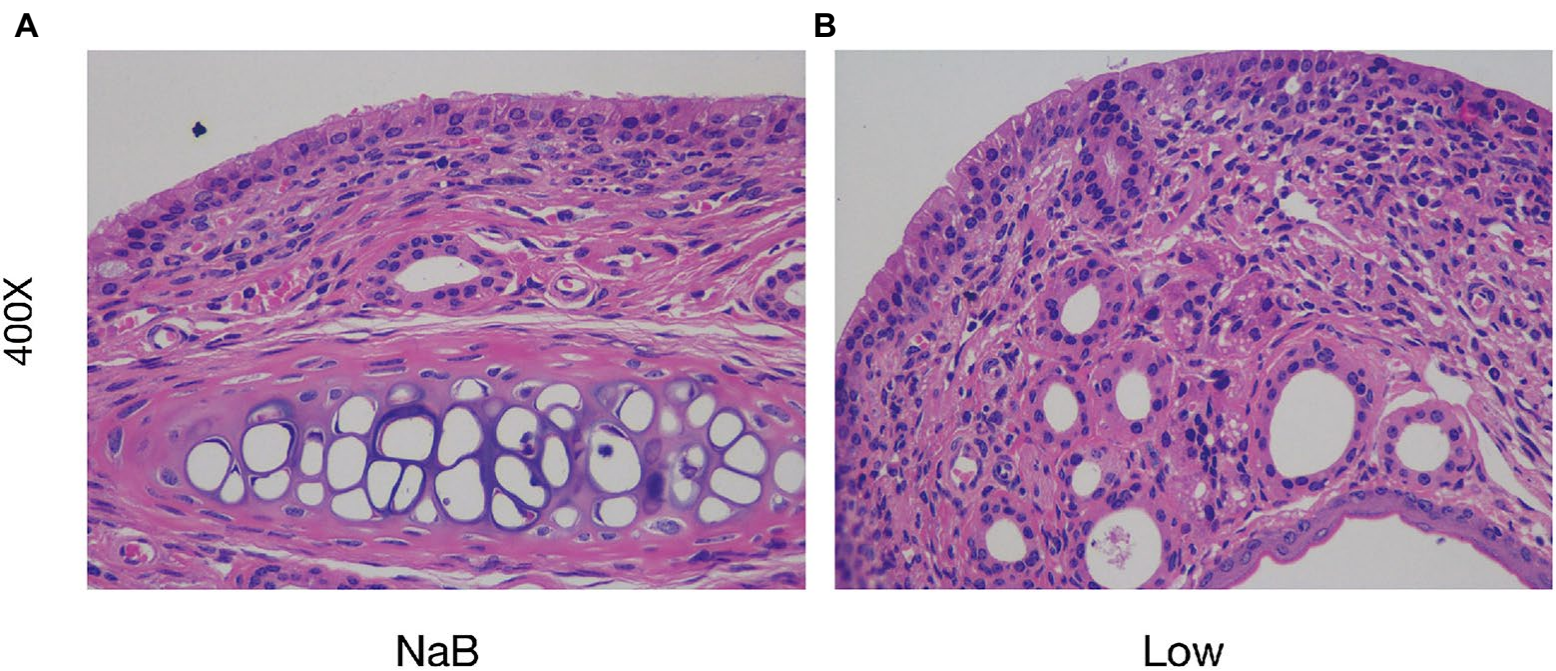
Sodium butyrate alleviates the infiltration of inflammatory cells in the nasal mucosa in the OVA-induced AR model. H&E staining of the nasal mucosa (400×). **(A)** the NaB group; **(B)** the Low group.

**Figure 13:**
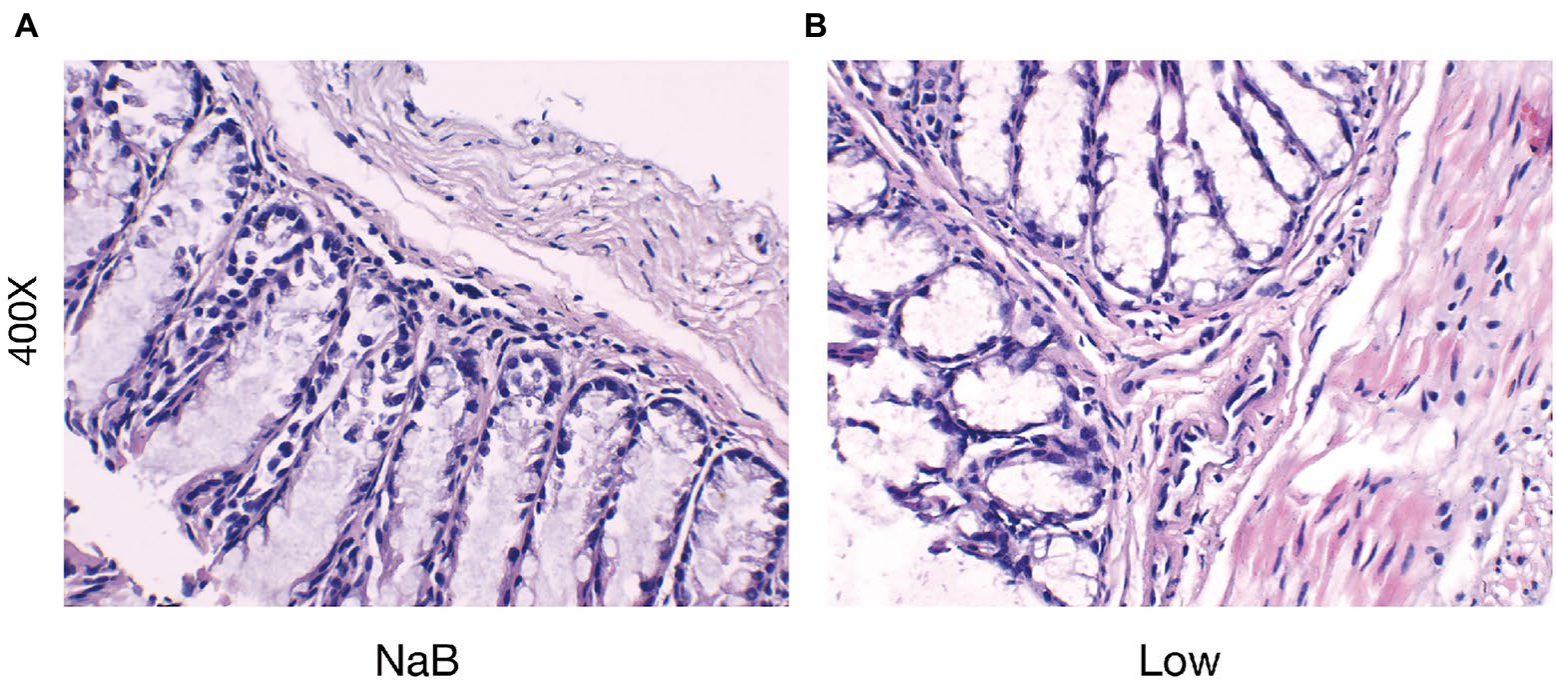
Butyrate reduced the severity of intestinal barrier inflammation and injury. H&E staining of colonic mucosa (400×) **(A)** the NaB group; **(B)** the Low group.

**Figure 14:**
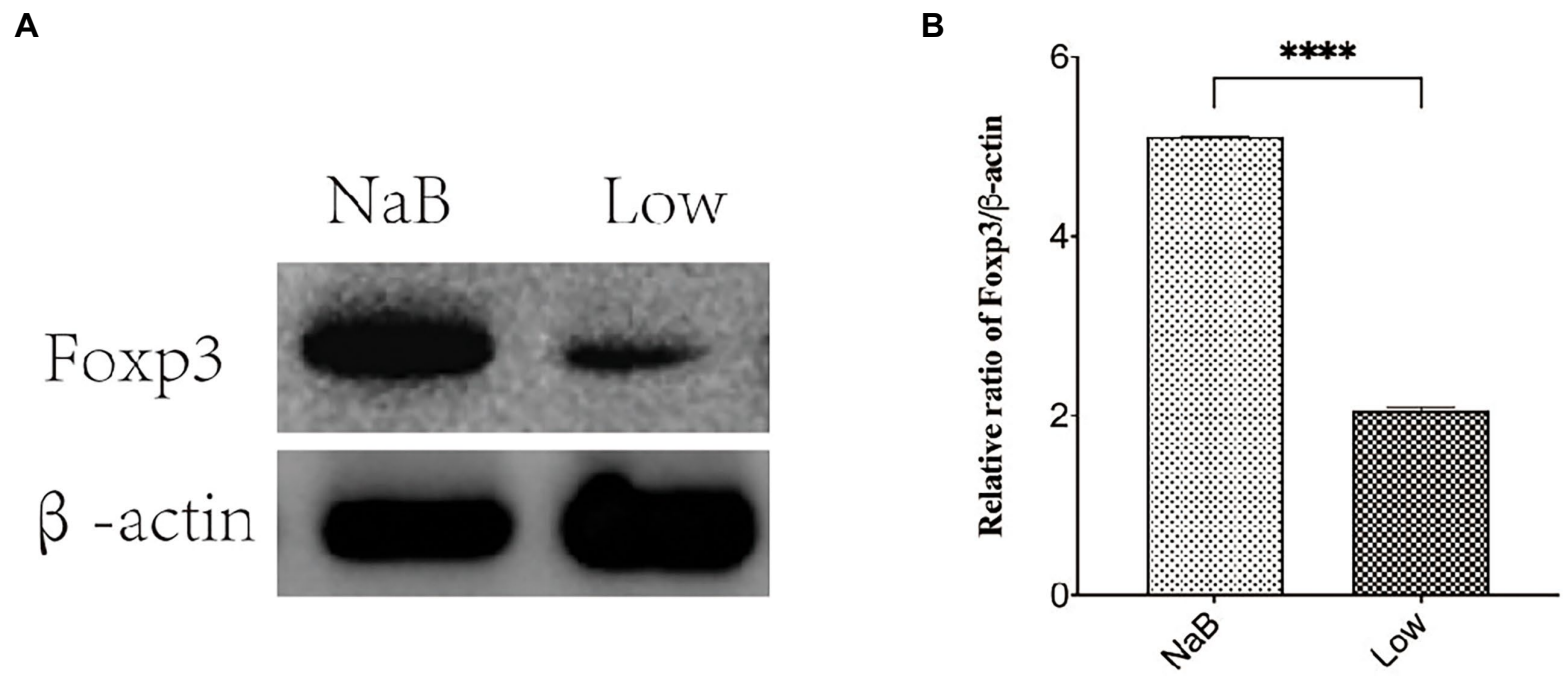
The protein expression of Foxp3 was detected by western blotting. **(A)** Protein banding. **(B)** Histogram. ^****^p < 0.0001.

Compared with the Low group, NaB treatment significantly reduced symptom scores (Supplementary Figure 2) and serum levels of OVA-sIgE (Figure 11A), total-IgE (Figure 11B), IL-4 (Figure 11C), and IL-5 (Figure 11D). In addition, these mice exhibited reduced inflammatory infiltration on H&E-stained sections of the nasal mucosa after NaB addition (Figure 12). In addition, IL-10 (Figure 11E) and TGF-β1 (Figure 11F) in the NaB group increased significantly compared with those in the Low group. Compared to the Low group, mouse intestinal tissues in the NaB group were more complete, with NaB alleviating the structural damage of colon mucosa caused by low dietary fiber (Figure 13). To further explore this mechanism, Foxp3 protein in the colon mucosa was analyzed by western blot. Foxp3 protein in colon mucosa was significantly upregulated after butyrate intervention (Figure 14), suggesting the expression level of Treg cells in the colon was upregulated. These findings suggest that NaB may alleviate the symptoms of AR by affecting intestinal mucosa and systemic immune homeostasis in mice.

## Discussion

To preliminarily explore the mechanism of vancomycin-induced gut microbiota dysbiosis in AR, a gut microbiota dysbiosis model was established in young mice (4 weeks old) treated with vancomycin, and the OVA-induced AR model was established in adult mice (6 weeks old). The results show gut microbiota dysbiosis occurred in mice treated with antibiotics, mainly, the diversity of gut microbiota (specific bacterial taxa) was significantly reduced. The allergic symptoms of AR mice with gut microbiota dysbiosis were significantly aggravated. At the same time, mouse serum OVA-sIgE, total IgE, IL-4, and IL-5 levels, along with H&E staining of nasal tissue, showed that gut microbiota dysbiosis dysregulates Th2 type allergic reaction and nasal inflammation in AR mice, which is consistent with previous studies of other allergic diseases (Yang et al., 2019; Kim et al., 2020). It is reported that infants with early gut microbiota dysbiosis are more likely to suffer from AR in adulthood (Zimmermann et al., 2019), but the corresponding molecular mechanism is not precisely understood.

In the present study, it was noted that the Chao and Shannon indexes were significantly decreased in the Van group as compared to the AR group. Moreover, PCA results showed gut microbiota from the Van group was distinguished from the AR group in the PCA plot. These results indicated a successful vancomycin-induced gut microbiota dysbiosis model was established. In this work, the proportion of gut microbiota being positively or negatively correlated with the severity of AR is shown. The relative abundance of Firmicutes and Bacteroidetes in the feces decreased significantly, while the relative abundance of Proteobacteria increased significantly. Agus et al. (2016) found the shift to Proteobacteria as the dominant flora is related to increased inflammation. Proteobacteria is a gram-negative bacterium, and its outer membrane contains many lipopolysaccharides (LPS), one of the most effective inflammatory inducers. Many studies show that LPS is significantly associated with the aggravation of AR symptoms (Bae et al., 2017; Iwasaki et al., 2017; Hu and Li, 2018). In addition, Proteobacteria has also been associated with many inflammatory regulations in some diseases, including asthma and atopic dermatitis (Noda et al., 1980; Reuschel et al., 2019; Wu et al., 2020). In this study, the increase in the proportion of Proteobacteria in the gut reflects the gut microbiota dysbiosis in mice after vancomycin intervention, which is also an essential factor leading to the aggravation of AR. In addition, this study found that, compared with the control group, the proportion of Firmicutes/Bacteroidetes increased in the AR and Van groups, with the Van group being higher than the AR group. Studies have shown the change in intestinal immune activity is related to an increased Firmicutes/Bacteroides ratio (Hou et al., 2021). The above results suggest that vancomycin-induced gut microbiota dysbiosis may be achieved by adjusting the proportion of gut microbiota. The change in the balance of dominant flora may be related to aggravation of nasal inflammation caused by vancomycin intervention.

Through Spearman association analysis, this study shows that vancomycin aggravates AR by reducing specific bacteria taxa, including SCFA-producing bacteria. The Van group had reduced genera, such as *Ruminococcus* and *Lactobacillus*, and were significantly positively correlated with fecal SCFAs. *Ruminococcus* plays a crucial role in metabolism. It obtains nutrients by breaking down host intestinal dietary cellulose, is one of the bacteria that produce SCFAs, and stabilizes the intestinal barrier, thus alleviating allergic diseases (Tun et al., 2017; Zhuang et al., 2021). This study found a significant decrease in *Ruminococcus* following gut microbiota dysbiosis and a significant, negative correlation in fecal SCFAs, suggesting that vancomycin-induced gut microbiota dysbiosis may aggravate AR by decreasing *Ruminococcus* and subsequently decreasing SCFAs. Antibiotic treatment can lead to consumption of steady-state microbiota related to protective functions, including *Lactobacillus*. *Lactobacillus* is gram-positive bacteria, which can protect the host from the potential invasion of pathogens. In addition, it can provide a source of nutrition for the host to metabolize lactic acid, acetate, and other end products, maintaining human health in many ways. Brusilovsky et al. showed that dysbacteriosis caused by antibiotic treatment reduces the abundance of *Lactobacillus* and aggravates type 2 inflammation. In addition, Durack et al. (2018) showed that supplementation with *Lactobacillus* temporarily altered delayed gut microbiota development in infants at high risk for asthma, possibly associated with increased levels of 4-acetylaminobutyric acid, a precursor of microbial SCFA biosynthesis. Here, this study shows that vancomycin treatment may consume *Lactobacillus*, reducing SCFAs and then aggravating AR.

In contrast, the increased floras in the Van group, such as *Escherichia-Shigella* and *Klebsiella*, were significantly negatively correlated with SCFAs in feces. *Escherichia-Shigella*, *Klebsiella*, and other gram-negative bacteria can produce many LPSs, resulting in an excessive inflammatory response (Goltermann et al., 2022). Liu et al. found that *Escherichia-Shigella* had significantly higher relative abundances in the AR group than in the healthy control group (Liu et al., 2020). These results suggest that vancomycin-induced gut microbiota dysbiosis may lead to the expansion of harmful bacteria, such as *Escherichia-Shigella* and *Klebsiella*, limit the production of SCFAs, aggravate the destruction of the intestinal mucosal barrier, and worsen AR.

SCFAs mediate the communication between gut microbiota and the host immune system (West et al., 2015), regulate the function of Treg cells, and maintain gut integrity as well as immune homeostasis(Wu et al., 2021), which affects the balance between pro-and anti-inflammatory cytokines in AR mice. These experiments suggest a significant reduction of SCFAs may be the key factor affecting vancomycin-induced gut microbiota dysbiosis in the AR model. However, to confirm the role of SCFAs in AR and exclude other possible confounding factors, a further control experiment was set up to study the effect of butyrate on AR mice fed a low dietary fiber basal diet. It was found for the first time in a mouse model of AR that butyrate feeding significantly increases Foxp3 protein levels in the colon, suggesting enhanced differentiation of Tregs in the colon mucosa. It is well-known that Treg cells produce anti-inflammatory cytokines, such as TGF-β1, and IL-10, to exert immune tolerance mechanisms (Zhu et al., 2010). Butyrate feeding increased serum levels of IL-10 and TGF-β1, which is consistent with Wang et al.’s study (Wang et al., 2016, 2020). This study suggests that butyrate may improve AR by upregulating Tregs in colon mucosa and increasing serum IL-10 and TGF-β1 levels to exert immune tolerance and inhibit allergic reactions. Kim et al. (2018) showed that Tregs balance in the colon may reduce symptoms of atopic dermatitis in mice, which is consistent with these results. In addition, butyrate intervention may alleviate inflammatory damage of colonic mucosa, which may be related to butyrate as an energy material of colon mucosa, strengthening the nutrition of colon mucosal cells and inhibiting local mucosal inflammation (Zhang et al., 2021; Peng et al., 2022). The specific mechanism needs more in-depth research.

Interestingly, our experiment found SCFAs increased in the feces of AR mice, which is consistent with mice with food allergies (Andreassen et al., 2018). It is possible, however, that different experimental designs explain the contrasting observed results, since the current SCFA levels were determined in animals after sensitization, while the protective effects were noted in animals whose microbiome composition or SCFA status were altered before immunization (Cait et al., 2018). In addition, different experimental schemes, mouse strains, sampling location, and time may also have affected the results of this study. This topic requires specifically designed studies to examine this question in further detail.

Due to the limitation of time and funds, it was not observed whether SCFAs could alleviate the symptoms of vancomycin-induced gut microbiota disturbances in AR mice. In addition, the content of SCFAs in serum and nasal mucosa were not detected. Further studies are needed to investigate changes in the intestine microbiome and SCFAs resulting from antibiotic treatment in the AR model.

## Conclusion

Vancomycin-induced gut microbiota dysbiosis increases the susceptibility and severity of AR in mice, which is significantly related to reduced SCFA-producing bacteria fecal SCFA levels or specific bacterial taxa. To the best of the authors’ knowledge, this study is the first systematic application of 16S rDNA technology and SCFA-targeted metabolomics technology to analyze the effects of vancomycin-induced gut microbiota dysbiosis in the AR model. In addition, NaB administration may be an effective treatment for low dietary fiber-induced symptoms and immune status in AR mice.

## Data availability statement

The data presented in the study are deposited in the SRA (Sequence Read Archive) repository (http://www.ncbi.nlm.nih.gov/Traces/sra), accession number PRJNA880782.

## Ethics statement

The animal study was reviewed and approved by the Institutional Animal Care and Use Committee (IACUC) of Fujian Medical University.

## Author contributions

ZC and QX designed the project, performed the experiment, drafted the manuscript, and they contributed equally. YL, YWe, and SH performed the experiment and collected the study data. YL and YWa analysed the data. LL and YX conceived the study, got administrative support, and they contributed equally. All authors contributed to the article and approved the submitted version.

## Funding

Funding was provided by United Fujian Provincial Health and Education Project for Tackling the Key Research (2019-WJ-25) and Natural Science Foundation of Fujian Province (2020J01981).

## Conflict of interest

The authors declare that the research was conducted in the absence of any commercial or financial relationships that could be construed as a potential conflict of interest.

## Publisher’s note

All claims expressed in this article are solely those of the authors and do not necessarily represent those of their affiliated organizations, or those of the publisher, the editors and the reviewers. Any product that may be evaluated in this article, or claim that may be made by its manufacturer, is not guaranteed or endorsed by the publisher.

## Supplementary material

The Supplementary material for this article can be found online at: https://www.frontiersin.org/articles/10.3389/fmicb.2022.1002084/full#supplementary-material

## Abbreviations

AR: Allergic rhinitis
SCFAs: Short-chain fatty acids
OVA: Ovalbumin
AD: Atopic dermatitis
SPF: Specific pathogen-free
NaB: Sodium Butyrate
GC–MS: Gas Chromatography–Mass Spectrometer
LDA: Linear discriminant analysis
Tregs: Treg cells
LPS: Lipopolysaccharide
PCA: Principal coordinate analysis
AA: Acetic acid
BA: Butyrate
PA: Propionic acid
IBA: Isobutyric acid
IVA: Isovaleric acid
VA: Valeric acid
CA: Caproic acid
ELISA: Enzyme-linked immunosorbent assay
H&E: Hematoxylin and eosin
