# Supplementary material for: Vancomycin-induced gut microbiota dysbiosis aggravates allergic rhinitis in mice by altered short-chain fatty acids
Source: Front Microbiol. 2022 Nov 1;13:1002084. doi: 10.3389/fmicb.2022.1002084 (PMC9687373; doi:10.3389/fmicb.2022.1002084)
Supplement: Supplementary file 1 [file Data_Sheet_1.pdf]

Supplementary Material

1 Supplementary Data

The original data of 16S rDNA sequencing for this study can be found by in the SRA (Sequence Read Archive) Database (http://www.ncbi.nlm.nih. gov/Traces/sra), which can be searched by "SRP397881".

2 Supplementary Figures and Tables

2.1 Supplementary Figures

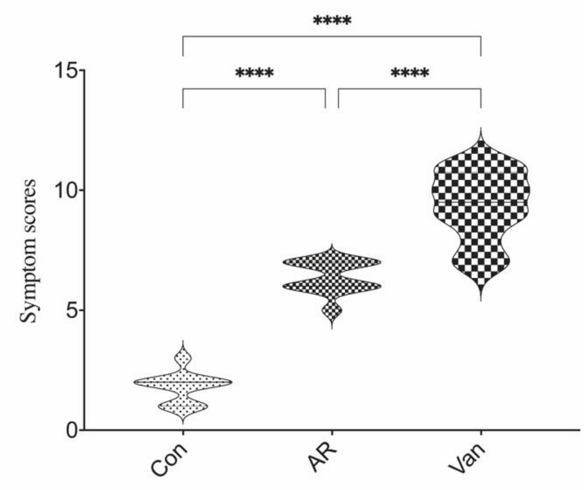

Supplementary Figure 1. Symptom scores of mice in the Control, AR, and Van groups. $*p < 0.05$, $**p < 0.01$, $***p < 0.001$, $****p < 0.0001$.

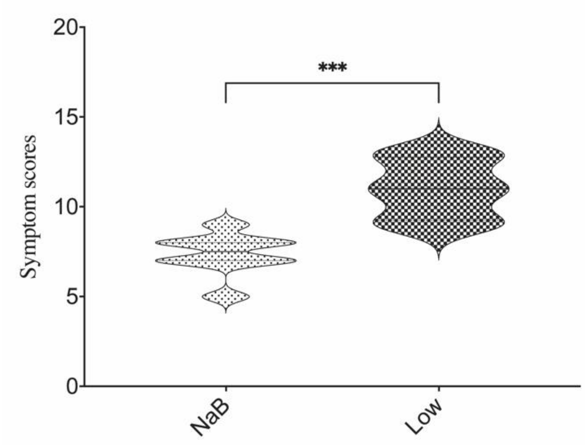

Supplementary Figure 2. Symptom scores of mice in the NaB and Low groups. $*p < 0.05$, $**p < 0.01$, $***p < 0.001$, $****p < 0.0001$.

2.2 Supplementary Tables

Groups / Elements	Low dietary fiber group (Low)	3% butyrate+Low dietary fiber group（NaB）
casein	0.7	0.7
Cystine	0.009	0.009
corn starch	2.5785	2.5785
Maltodextrin	0.775	0.775
sucrose	0.5	0.5
Soybean oil	0.2	0.2
Choline Bitartrate	0.0125	0.0125
Ain-93 mineral mixture	0.175	0.175
Ain93 vitamin mixture	0.05	0.05
Butyric acid	-	0.15
Total	5 kg	5 kg

Unit: Kilogram(kg)

Supplementary Tables 1: Low dietary fiber feed and low dietary fiber feed plus NaB feed were provided by the Xiao Shu You Tai（Beijing）Biotechnology Co., Ltd.

Symptom	Degree	Score
Nasal itch	Gently scratch the nose	1
	Severely scratch the nose	2
Sneeze	1-3	1
	4-10	2
	>10	3
Runny nose	Flow to the anterior nostrils	1
	Beyond to the anterior nostrils	2
	Flow all over the face	3

Supplementary Tables 2: Nasal symptom score scale. Nasal symptoms were assessed using the above scoring criteria: 1 point for sneezing 1-3 times during a 30-min observation, clear nasal flow to the anterior nostril and occasional scratching of the nose with a single front limb; 2 points for sneezing 4-10 times, clear nasal flow beyond the nostril and scratching of the nose with both front limbs or continuous scratching; and 3 points for sneezing more than of 10 times, clear nasal flow to the face. After the last stimulation, each animal was observed for 30 min, and the three parameters were recorded and quantified. A total score more than of 5 points indicated that the model was successfully established.

3. Other supplementary materials

The analysis and statistics of the metagenomic data:

The pair end (PE) double end sequence data obtained by Miseq sequencing is subjected to quality control processing on the measured Fastq data, and finally high-quality FASTA data is obtained.

Specific parameters are as follows:

1) Quality control of Fastq data was performed using trimmatic and pear. For trimmatic, a sliding window strategy is adopted, with a window size of 50bp, an average quality value of 20, and a minimum reserved sequence length of 120. Pear is used to remove sequences with N;

2) Flash and pear are used to merge the sequences at both ends according to the overlap relationship of PE. The minimum overlap is set at 10bp and the mismatch rate is 0.1 to obtain the fast sequence;

3) According to the known database, the chimera of FASTA sequence is removed by uchime method. For the unknown database, the self alignment (denovo) method is used to remove it, and the unqualified short sequence is removed at the same time.

Software: trimmatic (v0.36), pear (v0.9.6), flash (v1.20), vsearch (v2.7.1)
